# Deciphering culturally-coded institutional responses to COVID-19 adversity and anti-Asian hate in higher education

**DOI:** 10.3389/fpsyg.2025.1675867

**Published:** 2025-09-12

**Authors:** Shu-wen Wang, Janet Chang, Laurel R. Benjamin, Xueting Ni, Erin Walsh

**Affiliations:** ^1^Department of Psychology, Haverford College, Haverford, PA, United States; ^2^Department of Psychology, West Chester University of Pennsylvania, West Chester, PA, United States

**Keywords:** COVID-19, culture, higher education, discrimination, Asian American, independence, interdependence

## Abstract

**Introduction:**

Cultural values and belief systems are reflected in gateway contexts in societies, including educational settings. Yet, little is known about how values and norms are instantiated in higher education messages and how they may vary across cultural contexts during a global public health crisis. In this cross-cultural qualitative study, we examined cultural values and norms embedded in institutional messages at the early outset of the COVID-19 pandemic, exploring between-culture variations using an independence-interdependence framework. We also explored whether US higher education institutions addressed anti-Asian hate.

**Methods:**

We coded and analyzed early institutional announcements addressing the COVID-19 pandemic from the top 100 US universities and liberal arts colleges as well as 20 universities in China.

**Results:**

Thematic analysis revealed cultural similarities in Chinese and US institutional emphases on following political and medical authorities and collaboration. US institutions stressed support for students, “future as uncertain” appraisals, assurance of academic success, and validation of students’ emotions in line with soft independence. In contrast, Chinese universities promoted compliance, moral duty, and individual responsibility for a collective problem in line with interdependence. Few US institutions acknowledged Asians/Asian Americans as being targets of racial bias or discrimination, yet some announcements also implicitly linked COVID-19 with China or Asia (e.g., travel warnings and origin statements).

**Discussion:**

Findings illuminate the cultural patterning of norms, values, and priorities in different contexts in response to the same global event and demonstrate both the invisibility and hypervisibility of Asians/Asian Americans during a racialized global pandemic.

**Public significance statement:**

Select higher education institutions in the US and China emphasized political/medical authority and collaboration in announcements addressing the COVID-19 pandemic. US institutions tended to focus on independent needs and norms (student support, emotional validation, assurance of academic success) and framed the future as being uncertain, while Chinese universities stressed interdependent messages about compliance as well as moral duty and individual responsibility for a collective problem. Most US institutions overlooked Asians/Asian Americans as being potential targets of racial bias or discrimination in the context of the pandemic, yet some institutions implicitly linked COVID-19 to China or Asia.

## 1 Introduction

Models of self and agency are rooted in prevailing cultural values and belief systems, which are reflected in gateway institutions and contexts in societies across the globe. These cultural models are shared among individuals and reflected in daily life and practices in societies, with the framework of independence-interdependence being among the most dominant theoretical approaches to understanding cultural variations and orientations toward the self (Markus and Kitayama, 1991, 2010). Prevalent in WEIRD (Western, Educated, Industrialized, Rich, and Democratic) contexts (Henrich et al., 2010), like the United States, Western Europe, and Australia, values and norms ingrained in independence highlight the centrality of the self, autonomy, uniqueness, and freedom of self-expression (Markus and Kitayama, 1991; Triandis, 2018). In contrast, interdependent values and norms are common in Africa, Latin America, and Asia. Interdependence is often framed in terms of prioritizing the needs of close others, preserving group harmony and bonds, and viewing the self as embedded in social relationships (Markus and Kitayama, 1991; Triandis, 2018). In particular, Confucian values undergird a greater emphasis on conformity, obedience, and social hierarchy in East Asian cultures in comparison to uniqueness and self-differentiation in Western cultures (Kim and Markus, 1999) To date, a growing but small number of studies have examined how these cultural mandates of independence and interdependence are instantiated in higher education institutional settings (Chang et al., 2020; Stephens et al., 2012). Less is known regarding the ways in which cultural norms and values are reflected in institutional messages and how they may differ across cultural and educational contexts, especially in response to unique shared events such as a global pandemic. One exception is a recent study by O’Shea et al. (2022) that used crisis management frameworks to examine publicly available communications tracked over 6 months at 27 institutions in the US, China, and Canada. The authors found that Chinese institutions explicitly promoted compliance with the government and solidarity as a community, whereas US institutions demonstrated particular attentiveness to the impact of cancelation of athletic events and commencement ceremonies.

Culture shapes not only individuals and societies but also the psychological experience of the coronavirus disease 2019 (COVID-19) pandemic. In December 2019, the first cases of the virus were reported in China. The first US case of COVID-19 was reported on January 21, 2020, and the World Health Organization announced a global health emergency on January 30, 2020 (Keni et al., 2020). The COVID-19 pandemic fundamentally disrupted, upended, and altered daily routines, travel, educational and socioeconomic systems, social relationships and interactions, and ways of being. The consequences of the pandemic extended to all levels of individual, community, national, and global functioning, with well-documented impacts on subjective well-being and coping processes (Zacher and Rudolph, 2021), with evidence of additional cultural shaping of coping processes in the early stages of the pandemic (Benjamin and Wang, 2024). Gateway institutions, like universities and colleges, had to pivot to remote modalities and to implement institutional changes due to the outbreak and subsequent quarantine and governmental restrictions. The racialization of the COVID-19 pandemic also perpetuated racist stereotypes about Chinese people, heightening fear and hostile behaviors toward Asians/Asian Americans in the US (Cheah et al., 2020; Chen et al., 2020). It is within this context that we sought to examine the specific content and processes of culture during a global public health crisis by coding and analyzing higher education institutional announcements.

Cultural models of self are not mere properties of individuals that reside in the head; they have a psychological basis, but they are also enacted by individuals and institutions and manifest in structures, social practices, and products (Adams and Markus, 2004; Snibbe and Markus, 2005). Yet, research has focused much less on cultural products (e.g., advertisements, texts, and other collectively shared cultural representations) that foster and uphold different cultural norms (Cohen, 2007; Lamoreaux and Morling, 2012; Morling and Lamoreaux, 2008). Cultural products, like institutional and political messages, reflect both the psyche and sociocultural context—consequential to both the self and society at large. For instance, prior studies suggest media messages and political rhetoric may contribute to the “othering” of perceived outgroups (Eichelberger, 2007; Reny and Barreto, 2020).

Culture refers to consensually shared meanings that shape the self and influence behavior. In WEIRD contexts, norms aligned with independence encourage individuals to assert uniqueness, exercise personal choice, and prioritize personal needs and preferences, whereas norms aligned with interdependence focus on relatedness and encourage accommodation, adjustment, and orientation toward ingroup goals and the community (Markus and Kitayama, 1991; Morling et al., 2002). In the United States, higher education settings are institutional cultures that tend to be centered on independence, and specifically a form of soft or expressive independence that is more common in middle-and upper-class cultural contexts (Chang et al., 2020; Kusserow, 2012; Stephens et al., 2012). Soft independence emphasizes cultivated growth to reach one’s potential, in addition to influence, self-expression, and self-differentiation, whereas hard independence (more characteristic of working-class cultural contexts) emphasizes self-reliance, resilience, and emotional toughness (Chang et al., 2020; Kusserow, 2012). Thus, we aimed to examine cultural norms and values reflected in higher education announcements at the early outset of the COVID-19 pandemic.

After the start of the COVID-19 pandemic, prejudice and discrimination (i.e., derogatory and hostile attitudes and behaviors) against individuals of Asian descent increased in the US and other Western countries (Cheah et al., 2020; Chen et al., 2020; Jeung et al., 2021). Analyses of the online social media platform Twitter (now X) showed significant increases in anti-Asian sentiments in connection with the hashtags “COVID-19” and “Chinese virus” between March 9 and 23 in 2020 (Hswen et al., 2021), and over 11,000 reports of hate incidents targeting Asians/Asian Americans in the US were made to the Stop Asian American Pacific Islander (AAPI) Hate coalition after March 2020 (Stop AAPI Hate). Not surprisingly, research has found that US Asians (compared to US Whites) have experienced a disproportionate mental health impact due to COVID-19 related discrimination (Wu et al., 2021; Lee and Waters, 2021). This surge in anti-Asian hate takes place within a lengthy history of discrimination and violence against Asian Americans, perpetuated by US policy (e.g., Chinese Exclusion Act of 1882; Takaki, 1998) and prevailing societal practices (e.g., racial denigration and misperceiving Asian Americans as foreigners; Huynh et al., 2011). Racialized scapegoating of Asian Americans has a long-standing US history, with anti-Asian rhetoric and violence escalating during periods of political, economic, and social duress [e.g., internment of Japanese Americans during World War II; racialization of the 2003 severe acute respiratory syndrome (SARS) epidemic; Eichelberger, 2007; Takaki, 1998]. The racialization of COVID-19 (e.g., “Wuhan or Chinese virus,” “kung flu”) evokes the centuries-old “Yellow Peril” racist trope that renders those perceived to be Asians as a vile and dangerous foreign evil (Chen et al., 2020; Takaki, 1998). Although Asian Americans have increased visibility and presence in terms of numeric representation related to US population growth (Vespa et al., 2018) and college enrollment (National Center for Education Statistics, 2021), Asian Americans are marginalized and understudied, and research on Asian Americans is underfunded (Đoàn et al., 2019; Yi, 2020).

In sum, we sought to explore both cultural variation and similarity in the norms and values of higher education announcements, with attention to institutional messaging on anti-Asian hate, at the early outset of the COVID-19 outbreak.

### 1.1 The current study

The current cross-cultural qualitative study used naturalistic data (i.e., drawn from the phenomenon’s natural setting) and employed thematic analysis (Braun and Clarke, 2006, 2022), a methodology that is well-suited for investigating and illuminating cross-cultural patterns in qualitative data drawn from their local contexts. We explored the following research questions:

1. What prevailing cultural norms and values are found in online public-facing announcements posted by higher education institutions in the US and China at the outset of the COVID-19 outbreak?
2. What are cross-cultural variations in cultural mandates reflecting independence and interdependence (e.g., cultural imperatives connected to threat, vulnerability, individual/collective responsibility, and suggested actions)?
3. What kinds of messages do US higher education institutions convey about anti-Asian xenophobia and discrimination at the outset of the racialized context of the pandemic?

We gathered, coded, and analyzed public-facing higher education announcements about their institutional response to COVID-19 in the US and China. This methodological approach is consistent with past research demonstrating that representations of cultural values and norms manifest in cultural products “outside the head” (Morling and Lamoreaux, 2008), including song lyrics (Snibbe and Markus, 2005), children’s books (Tsai et al., 2007), school textbooks (Imada, 2012), and the like. Studies on the culture of education are overall sparse and have tended to rely on self-report survey data (e.g., asking university administrators to report on institutional expectations; Stephens et al., 2012). While there is value in using cultural experts, cultural contexts are also integral in the transmission of values and norms in higher education settings.

We conducted thematic analysis, an approach designed to shed light on both observable (manifest) and underlying (latent) content by recognizing, categorizing, analyzing, and revealing patterns across qualitative data (Braun and Clarke, 2006, 2022). Thematic analysis can be inductive (“bottom up” or data-driven) or theoretical (“top down” or analyst-driven) in the process of coding data (Braun and Clarke, 2006, 2022). A notable strength of thematic analysis is deepening comprehension of textual meaning (Neuendorf, 2018). As such, cultural products and qualitative methods serve as an important methodological means—providing depth of understanding of the dynamic relationship between cultures and psychological phenomena.

## 2 Methods

### 2.1 Sample of higher education institutions

The sample included 50 US national universities, 50 US liberal arts colleges, and 20 Chinese universities. Using the US News and World Report rankings, we identified the top national universities (*n* = 50) and top liberal arts colleges (*n* = 50) in the US. If universities or colleges were tied in ranking, we included those institutions that were tied until we reached a total count of 50, respectively. Prestigious universities in China (*n* = 20) were selected based on Project 985 and Project 211, two 1990s-early 2000s governmental programs that identified top universities based on set criteria indicative of norms and priorities in China. These programs were identified via cultural consultation and are commonly referenced in Chinese media as reflecting the top universities, and our sample widely reflects 16 of 23 Chinese provinces. We decided upon the 20 Chinese universities based on which ones had COVID-19 institutional announcements that were available online at the time of data collection (March–April 2020), and also because of the brevity and government-directed uniformity (described further below) in the announcements from Chinese universities. Further examination of a larger selection of Chinese institutional announcements was contraindicated because we reached saturation with the sample of 20 Chinese institutions. We had collected enough rich data to identify consistent patterns and recurring themes, from which we developed a comprehensive understanding of the phenomena, with new data failing to provide any further insights. A systematic review of qualitative research by Hennink and Kaiser (2022) concluded that saturation can be reached by samples of 4 to 8 (focus groups) and 9 to 17 (interviews), especially when considering the relatively homogeneous sample and the specific narrow objective of the study.

### 2.2 Procedure

The research team procured announcements about the COVID-19 outbreak and resulting shift to remote instruction that were posted on higher education institutional websites (US announcements dated March 1, 2020 through March 19, 2020; Chinese announcements dated January 26, 2020 through February 2, 2020). Data collection took place March through April 2020. We archived publicly obtained announcements posted online by the US and Chinese higher education institutions, selecting the first comprehensive announcement issued by each institution (President/Provost/administration) about their COVID-19 response and communicated to faculty/students/staff. Chinese announcements were translated into English by co-author XN, who is a Mandarin-English native bilingual. Institutional announcements varied in length between the two countries. On average, announcements from US institutions were 1,390 words (1,331 words for liberal arts colleges, 1,449 words for national universities), whereas translated Chinese institutional announcements were much shorter in length (293 words; 226 words excluding three extreme outliers). In short, Chinese announcements were on average only about a fifth of the length of US announcements.

It is important to note that there was a high degree of uniformity in the use of key phrases across nearly all Chinese announcements, due to national guidance or mandates given the political structure of China. In particular, at least one of three sentences appeared verbatim (or a close variant) in over half of all Chinese announcements: “Consciously preventing and controlling the epidemic is not only related to the safety and health of your life and that of everyone around you, but is also the legal responsibility and obligation of every citizen,” “Everyone of us has a responsibility to prevent and control this epidemic,” and “Preventing and controlling pneumonia outbreaks of the new coronavirus infection has become a top priority.” When considering the overall brevity of the Chinese announcements and the amount of similarity therein, we reached saturation quickly with a sample of 20 announcements.

### 2.3 Qualitative coding and thematic analysis

All five authors were members of the research team and came from diverse cultural backgrounds (Taiwanese, Chinese American, White American, and Vietnamese). All were involved in codebook development and an initial review of institutional announcements. Three research assistants (co-authors LB, XN, EW) engaged in coding, while a fourth research assistant served as an internal auditor throughout coding. First author SW supervised and audited coding and analyses, including how codes were ascertained and evidence used in support of codes and results, and second author JC conducted a final review of codes and results, and shared observations and insights.

A database of archived institutional announcements was created for coding followed by codebook development, coding, auditing, discussion and identification of themes, and a final review. The three primary coders and the two internal auditors read all institutional announcements, contributed to the development of all codes, and examined all codes. Congruent with a thematic analysis approach, the qualitative process involved initial coding followed by manifest and latent coding, with the goal of discovering themes (Braun and Clarke, 2006, 2022; Ryan and Bernard, 2003). Coders indicated the presence of a code (1) or absence of a code (0). Initial codes were generated by examining the raw data in the announcements. The research team derived categories of codes, identified discrete codes, and developed definitions and exemplars of codes based on textual analysis (Ryan and Bernard, 2003). Coders explored similarities and differences between institutional announcements to identify broader categories and subcategories of codes. For instance, *Compliance, Collaboration, Individual Responsibility,* and *Moral Duty* were discrete codes in the broader category “Responsibility.” An iterative, constant comparative method was used to generate associations between codes and expand on and finalize key codes (Fram, 2013). This process contributed to the development of an organized understanding of patterns and a narrative about emerging themes. The team developed a working understanding of overarching patterns across broad categories and uncovered similarities and differences in discrete codes across types of institutions and different cultural contexts.

To promote rigor and thoroughness in coding and thematic analysis, the research team engaged in open discussion of codes, provided feedback to each other drawing on evidence, shared detailed observations, and resolved coding inconsistencies, and auditing was used to promote reflexivity (Morrow, 2005). Intercoder reliability was determined by calculating percent agreement for final codes averaged across the three coders; average coder agreement was high (96%). Regular team discussion, internal auditing, and a final review served to ensure the integrity of codes and verify insights that emerged (Elo et al., 2014; Stahl and King, 2020). At the end of this process, the research team identified and elaborated on dominant themes, with consideration of similarities and differences across types of institutions and cultural contexts.

## 3 Results and discussion

Thematic analysis revealed cultural similarities and differences between higher education institutions in the US and China in the ways they addressed the COVID-19 pandemic. In particular, the patterns highlighted cultural differences between US and Chinese institutions in the kinds of independent and interdependent values invoked to motivate collective action and to recognize specific needs and norms. Second, the results revealed a paucity of US higher education institutions simply recognizing, much less denouncing, the occurrence of anti-Asian hate and discrimination, while also illustrating implicit biases in linking COVID-19 with China or Asia.

### 3.1 US-China cultural similarities

#### 3.1.1 Following political and medical authorities

Cross-culturally, we found that US and Chinese institutions had a shared emphasis on following the guidance of political and medical authorities (79%). This was not surprising given the announcements pertained to a global public health emergency. US national universities and liberal arts colleges (76 and 78%, respectively) tended to refer to the guidance of the CDC (Centers for Disease Control and Prevention), State Department, and other public health experts. Universities in China referred to the Central Leading Group for Coronavirus Prevention, the Ministry of Education, local requirements, and provincial authorities. Nearly all Chinese universities stressed following political and medical authorities (90%).

#### 3.1.2 Collaboration and collective action

Many higher education institutions across the US and China placed importance on *Collaboration* (53%), but there were nuances that are described in the sections below. US liberal arts colleges (64%) and national universities (44%) tended to emphasize working together (“your collaborative spirit,” “coming together to work out solutions that are in the best interest,” etc.) and recruiting the input or assistance of members of the campus community. Similarly, Chinese universities (50%) stressed collaborative efforts (“work together,” “unite our efforts”) as members of the community. Together, these cultural similarities underscore the necessity of policies to guide institutional action and viewing individuals as members of communities being called upon for collective action in response to a global public health crisis.

### 3.2 US-China cultural differences

#### 3.2.1 Egalitarian collaboration as partners vs. vertical collaboration and compliance

There were cultural differences in the norms and values reflected in the patterns that emerged. Chinese universities tended to convey interdependent messages about *Compliance* (85%). Although we saw elements of collaboration in both Chinese and US announcements, collaboration co-occurred with compliance in Chinese announcements, suggesting a hierarchical or vertical form of collaboration in which there is submission to a higher authority. This finding is consistent with a cultural orientation in which Confucian-rooted conformity (Kim and Markus, 1999) and respect for authority are highly valued (Shavitt et al., 2011). Compliance was most salient, with Chinese announcements presenting collaboration as a set of expectations seemingly rooted in patriotic fervor to rally Chinese citizens toward collective action. Chinese announcements tended to refer to the pandemic as a “battle” and urged citizens to “join hands” or “unite.” In promoting compliance, Chinese universities instructed students and staff to “obey” and “follow orders”; “should” and “must” language were often used to accompany directives about “requirements.” This is similar to O’Shea et al. (2022) that found an emphasis in Chinese higher education communications on obeying policy by central government, municipal government, and the Ministry of Education, often using military language.

We note that compliance is reflected in the simple fact (previously reported in the Method section) that there was a high degree of uniformity in the use of specific key sentences across the Chinese announcements (“Consciously preventing and controlling the epidemic is not only related to the safety and health of your life and that of everyone around you, but is also the legal responsibility and obligation of every citizen,” “Everyone of us has a responsibility to prevent and control this epidemic,” and “Preventing and controlling pneumonia outbreaks of the new coronavirus infection has become a top priority.”), demonstrating obedience to government guidance and further illuminating the nature of state-institutional relations in China. The amount of uniformity is especially compelling when considering the overall brevity of Chinese announcements (average 293 words vs. 1,390 words in US announcements); the announcements convey an (unmistakably) clear and (remarkably) concise message that illustrate how China’s political structure shapes institutional messaging during a global health crisis. Overall, this form of compliance illustrating vertical collaboration—consistent with interdependence—rarely emerged in US institutional announcements (6% liberal arts and 4% university).

Rather, US institutions tended to ask for collaboration from students, faculty, and staff—requesting community members’ active and voluntary partnership in achieving a common goal. Frequently conveyed across US institutional announcements, the sentiment of “we are all in this together” highlights everyone’s active role in addressing the situation, “succeeding together,” and “with your help.” For example:

“The College needs your wisdom and experience as we partner to make the [College] experience – if modified – possible, despite the conditions in which we must operate. We are all in this together, and will succeed together.” (U.S. institution)

In evoking partnership, US institutions tend to foster a more egalitarian environment in which each community member is important to the success of the shared mission. The findings are in line with independent norms that entail seeing individuals as equal in status (i.e., Markus and Kitayama, 1991, 2010). Language used included “invite” and “welcome” to garner individuals’ input and engagement, and gratitude was expressed for everyone’s partnership and contributions, highlighting individual agency in the collaborative process. For example, one institution expressed:

“I am profoundly grateful for what I know will be your steadfast commitment to students and your collaborative spirit that will enable us to rise to this occasion. Indeed, I am humbled by the expressions I have already heard from many of you about your willingness to try to do right by your students, even under these challenging circumstances.” (U.S. institution)

Similarly, another institution stated:

“I welcome the input of all members of our community as we consider the range of issues this unprecedented situation has created.” (U.S. institution)

Consistent with interdependent norms and values regarding duty to others, hierarchy, social roles, and collective action for the common good (Markus and Kitayama, 1991, 2010), the majority of Chinese announcements emphasized *Moral Duty* (60%) and *Individual Responsibility for a collective problem* (60%). *Moral Duty* referred to broader moral imperatives or principles that needed to be met, including societal and legal obligations, and *Individual Responsibility* highlighted the specific distinct role played by the individual to help solve a collective problem. For instance, one Chinese institution declared:

“Preventing and controlling the epidemic is the responsibility and duty of each and every citizen.” (Chinese institution)

The majority of Chinese institutions further explained:

“Consciously preventing and controlling the epidemic is not only related to the safety and health of your life and that of everyone around you, but is also the legal responsibility and obligation of every citizen.” (multiple Chinese institutions)

On the whole, Chinese announcements focused largely on interdependent values centered on duty, obligation, and deference to authority, underscoring a vertical form of collaboration (alongside compliance) that differed from the more egalitarian form of collaboration found in US announcements. The patterns attest to the differential emphasis on interdependence in Chinese announcements and independence in US announcements.

#### 3.2.2 US focus on independent needs and norms

In stark contrast, US institutions emphasized the importance of independent needs and norms by: identifying and providing support for specific student needs (*Informational Support* 88% and *Instrumental Support* 81%); through recognition of internal processes within the individual via attunement to emotional experiences (*Emotional Validation* 60%) and cognitive appraisals of the *Future As Uncertain* (72%); and last, addressing individuals’ goals by providing *Assurance of Academic Achievement* (61%).

##### 3.2.2.1 Support provision

Virtually all US institutions (liberal arts colleges and universities) provided some channel for student support. Most (88%) US institutions provided informational support (e.g., a FAQ or information hub where students could receive up-to-date information about the school’s changes as the virus progressed), as well as provided housing, financial support for travel, internet access, or other forms of instrumental support (81%) for students with extenuating circumstances. Additionally, some US institutions also referred students to emotional support services including college counseling and telehealth resources (*Emotional Support* 18%). Our findings dovetail with the results of another study that found financial and material support provision to be a central theme in COVID era higher education announcements in the US (O’Shea et al., 2022). By comparison, our data show relatively little expression of any kind of support provision (*Informational Support* 20%; *Emotional Support* 5%; *Instrumental Support 5%*) in Chinese announcements.

##### 3.2.2.2 Attunement to emotional states and “future as uncertain” appraisals

The majority of US institutions demonstrated attunement to community members’ potential emotional states (*Emotional Validation* 60%). These institutions frequently delineated different kinds of feelings that students could be having, such as worry, fear, and disappointment over lost experiences (e.g., athletic events). This dovetails with a large body of cultural research that has shown an emphasis on emotion identification and expression in the US, that emphasize influence and autonomy (Kang et al., 2003; Matsumoto et al., 2008) and prioritize maximizing positive and minimizing negative feeling states (Spencer-Rodgers et al., 2010). Sometimes, these announcements conveyed a desire to ease those negative feelings through compassion and empathy and/or even through claiming some responsibility (e.g., by apologizing) for the challenges and discomfort of COVID-19 disruptions. For example, one US institution conveyed:

“I am sorry to have to share such heartbreaking news … I especially feel for our seniors whose ‘senior spring’ is being severely disrupted.” (U.S. institution)

And another US institution expressed:

“Again, we recognize that these changes to the spring semester are disappointing and frustrating. We apologize for all of the difficulties and questions that they are creating for you and your loved ones.” (U.S. institution)

Similarly, US announcements frequently expressed cognitive appraisals of the *Future As Uncertain* (72%), calling upon community members to be adaptable and flexible in the face of rapidly changing and unpredictable circumstances. One institution stated:

“This has been, and will continue to be, a very fluid situation filled with a lot of uncertainty.” (U.S. institution)

Another appealed to its community members with the following:

“I ask that we remain flexible and also diligent, knowing that matters are changing rapidly.” (U.S. institution)

In a similar vein, the following announcement addresses the uncertainties of COVID-19:

“The novel coronavirus, COVID-19, presents our world, country and University with a unique public health challenge that is all the more disquieting because of the uncertainties associated with its spread.” (U.S. institution)

These findings characteristic of US higher education institutions align with the educational strategy of fostering a future time perspective, encouraging students to think about the future and their present and future goals, despite living in a world where the future is increasingly more uncertain (Morselli, 2013).

The US recognition of emotional distress and future uncertainty is in explicit contrast to Chinese announcements that rarely validated emotional experiences (5%) and never referred to appraisals of an uncertain future (0%). Scholars have found that East Asian beliefs rooted in Confucian values (naïve dialecticism) endorse the expectation of change and contradiction, versus the notion of change being linear and predictable found in Western analytic thinking (Spencer-Rodgers et al., 2010). We reason that the articulation of the future as uncertain and ever-changing may be unnecessary in Chinese contexts since this concept is already culturally syntonic with Chinese thinking, whereas US institutions may have specifically communicated this idea because it is dissonant with prevailing cultural thought.

##### 3.2.2.3 Ensuring academic achievement

Last, US colleges and universities explicitly addressed individual goals of academic achievement by expressing an institutional commitment to helping or “ensuring” that students will succeed (*Ensuring Academic Achievement* 61%). These announcements frequently listed specific actions taken by the institution, for example:

“[College] is committed to maintaining our daily operations, completing the semester, and ensuring that all students can fulfill their academic requirements as planned.” (U.S. institution)

Another institution articulated their commitment to student success during remote learning:

“[We] will do everything possible to ensure your continued success. [We] have been fully engaged in setting you up for success as you continue the semester remotely.” (U.S. institution)

In the present study, the tendency of US institutions to frame the future as being uncertain in conjunction with an emphasis on students’ assured success may serve to foster a future time perspective. Previous research has found that a future time perspective coupled with self-certainty (e.g., sense that one has the tools to persevere and achieve one’s goals) led to increased planned study hours and goal-focused action in students (Smith et al., 2014). US institutions promoting students’ self-efficacy in the face of an uncertain future may be particularly motivating to students in independent cultural contexts during troubled times, armed with the certainty that they have the tools and supports for personal success.

Rather than guaranteeing that students attain their goal of academic achievement, Chinese institutions, on the other hand, stressed to some degree *Productivity and Discipline* (20%), for example:

“I hope that the students take ample protection of themselves, actively maintain communication with counselors, head teachers, and class teachers, do a good job at independent learning, and strengthen physical exercise.” (Chinese institution)

There were also simple directives to work on academics:

“Students, please make the best use of your time at home to improve your studies.” (Chinese institution)

An emphasis on productivity and discipline during turbulent times is in accordance with Chinese cultural values that emphasize forbearance (Wei et al., 2012) and emotional self-control (Butler et al., 2007) in response to adversity, challenge, or stressors.

Overall, the US institutional announcements centered independent needs and norms that specifically aligned with soft independence (Chang et al., 2020; Kusserow, 2012; Stephens et al., 2012); a focus on cultivated growth to reach one’s potential (via intensive institutional support and ensured academic achievement), the recognition and validation of emotional experiences, and the explicit framing of the future as uncertain that may potentially enhance motivation and effort when coupled with provision of support and resources in an independent cultural context.

### 3.3 Invisibility of Asians and Asian Americans in the US

Very few US universities and colleges recognized Asians and Asian Americans as being possible targets of racial bias, stereotyping, or discrimination due to racialization of COVID-19 at the early outset of the pandemic. Overall, 9 out of 100 US institutions acknowledged any form of bias, stereotyping, or discrimination that occurred as a result of the COVID-19 pandemic. Among these institutions, only four explicitly recognized Asians and Asian Americans as targets of hate.

A small number of institutions (7 out of the 9 mentioned above) took a stance against hate or discrimination connected to the COVID-19 pandemic. In an explicit example of this denouncement, one university situated in the Western region of the US asserted:

“The fear of Coronavirus has resulted in incidents of bias and harassment against Chinese nationals, Chinese Americans, and people of Asian heritage here, and worldwide. Many members of our community, including our international students, faculty, and staff, may be dealing with stigma. Please uphold our commitment to antiracism.” (U.S. institution)

Furthermore, very few institutions directed students, faculty, or staff to campus resources to handle bias or harassment. In the following example from one Midwestern university, denouncement was followed by specific guidance:

“Racist behaviors or stereotyping in or outside of the classroom are not acceptable at [institution name omitted]. We encourage students who experienced harassment or discrimination to file a bias incident report. Employees may file a complaint with the Office of Compliance. We need everyone’s support during this challenging time and to treat each other with respect and kindness.” (U.S. institution)

In a similar vein, one Midwestern liberal arts college announced:

“At times during public health emergencies, individuals can unfortunately be subject to bias based on incorrect beliefs of connections between their perceived identity, their citizenship or visits to impacted locations and risks to health. If you need support or want to learn more about these issues, please contact the Multicultural Resource Center at [phone number omitted]. The Office of Equity, Diversity, and Inclusion [phone number omitted] may also be a helpful resource.” (U.S. institution)

Strikingly, the lack of attention to anti-Asian bias and discrimination took place alongside implicit biases that linked the novel coronavirus with China and, subsequently, may have racialized the virus. Announcements did this in two ways: by describing a geographic “origin” to the coronavirus (*Geographic Origin* 5%) and by mentioning travel warnings that prohibited travel to China (*Prohibited Travel* 14%). In doing so, Asians and Asian Americans were rendered both invisible (in terms of acknowledgements of anti-Asian bias and discrimination) and hypervisible (being linked with the coronavirus), reinforcing longstanding US narratives that view Asians and Asian Americans as perpetual foreigners posing threat to American civilization (Chen et al., 2020; Takaki, 1998). Given the specific positionality of Chinese international students in the US during this period of time, acculturation and identity conflicts (Jin et al., 2024) in addition to perceived discrimination due to COVID-19 (Jin et al., 2025) may have impacted health and well-being as well as the reintegration processes upon returning to China.

We note that our data capture a unique “slice” of institutional response at the immediate outset of the COVID-19 pandemic. Another study that tracked higher education announcements over a period of 6 months concluded that US announcements explicitly denounced racism, xenophobia, and discrimination (O’Shea et al., 2022). Yet, other studies noted institutional deprioritization of responding to anti-Asian hate (Castro Samayoa et al., 2023) and vagueness in responses that failed to specifically acknowledge targeted communities (Soltis, 2005), which align with our analysis.

In sum, we situated the US cultural emphasis on independent needs and norms within the broader context of implicit bias linking Asians and Asian Americans to COVID-19 while rendering anti-Asian hate and discrimination invisible. Our findings demonstrate how US cultural values conveyed through institutional announcements co-exist with the racialization of Asians and Asian Americans and anti-Asian racism, illuminating the interconnections between our qualitative themes.

## 4 General discussion

The present study revealed that the cultural mandates of independence and interdependence are enacted in different ways in the US and China. US institutions emphasized collaboration of an egalitarian nature and addressed independent norms that included supporting student needs, validating emotional experiences, framing the future as being uncertain, and ensuring student academic achievement. In contrast, Chinese university announcements focused on compliance to a set of expectations, a moral obligation to act in response to COVID-19, and individual responsibility to the collective. This is all consistent with a large body of research documenting cultural differences in independence and interdependence. However, cultural similarities between US and Chinese colleges/universities underscore the shared importance placed on guidance from political and medical authorities and members of institutional communities collaborating together, reinforcing the centrality of a global public health emergency in guiding institutional responses.

Our exploratory findings also attest to the larger issue of both invisibility and hypervisibility of Asians and Asian Americans in US higher education settings—their vulnerability as racialized subjects during a period of elevated anti-Asian hate made invisible, while their association with a racialized virus made hypervisible. These are not surprising findings because Asians and Asian Americans are perpetually invisible in the racial discourse in scientific research, higher education settings, and society at large (Tseng and Lee, 2021), and have been historically rendered ‘the other’ as a source of peril and threat in the US (Chen et al., 2020; Eichelberger, 2007; Takaki, 1998). Our analysis suggests that the initial response to COVID-19 in March 2020 by most US institutions we examined was guilty of both reflecting and transmitting the same cultural messages.

### 4.1 Limitations

The research presented here provided a rich cross-cultural analysis with novel insights and some important caveats. The most notable limitation of the study is the fact that we sampled the top universities and colleges in the US and China, and thus we did not examine other types of higher education institutions (e.g., community colleges and public regional universities). Independent and interdependent norms may differ across academic settings given student sociodemographic characteristics (Chang et al., 2020), and past research has shown that 2-year colleges tend to promote more interdependent institutional norms (Tibbetts et al., 2018). Additionally, we examined public-facing announcements posted to institutional websites in the beginning weeks of the COVID-19 pandemic (i.e., March 1, 2020 through March 19, 2020 in the US, January 26, 2020 through February 2, 2020 for China) to capture this unique window of time at the start of the pandemic, and did not take into account concurrent or subsequent communications that may have taken place through means accessible only to members of that institution (i.e., email, password-protected communication boards). However, we note that the public-facing nature of these announcements—posted to institutional homepages—reinforces their primary role as conduits of institutional values and norms openly broadcasted to people both within and outside the institution. Arguably, this renders these initial public responses more compelling as cultural products. Last, we did not examine how institutional messages may have changed or stayed consistent across time as events related to the COVID-19 pandemic unfolded. For example for US institutions, we note that the surge in anti-Asian hate and discrimination peaked after the March 2020 timeframe within which we identified the first comprehensive statement released by each US institution. Yet we note that the initial communication may represent a more direct and unadulterated reflection of institutional values and norms than those shaped by subsequent public discourse and events; for example, hate crimes tracking (i.e., by Stop AAPI Hate, n.d.). Altogether, we emphasize the important advantage of cultural products, such as public-facing institutional announcements, as being less vulnerable to self-report biases (Lamoreaux and Morling, 2012).

Cultural contexts and products are integral to the transmission of societal values and norms. The present findings showed how cultural patterns and differences stem from forces outside the head and out in the world, illuminating how institutional responses are shaped by cultural and social forces within local, national, and global contexts. Exploring the co-construction of culture through cultural products is vital to advancing and understanding cultural imperatives to be an independent and/or interdependent self embedded in larger sociohistorical contexts.

## Data Availability

The raw data supporting the conclusions of this article will be made available by the authors, without undue reservation.
